# Objective Refraction Status before and after Cycloplegia: From Childhood to Young Adulthood

**DOI:** 10.3390/vision8030051

**Published:** 2024-08-30

**Authors:** Karola Panke, Megija Jorova

**Affiliations:** Department of Optometry and Vision Science, University of Latvia, Jelgavas Street 1, LV-1004 Riga, Latvia

**Keywords:** cycloplegia, cyclopentolate, refractive error, autorefractometer, prevalence

## Abstract

This study aimed to evaluate the clinical information revealed after cycloplegia and assess how age and non-cycloplegic refractive status influence the classification of types of refractive error, as well as the relationship between age and cycloplegia-induced changes in the power of refractive errors. We analysed the records of 472 non-population-based ophthalmology practice patients aged 3–28 years (mean ± SD: 9.1 ± 4.6). Cycloplegia was induced with one drop of cyclopentolate 1% in each eye, and eye refraction was measured 30 ± 5 min later using an objective autorefractometer. Cycloplegia induced a clinically significant (≥0.50 D) hyperopic shift in the spherical equivalent of 60.2% of participants and a myopic shift in 1%, resulting in a 34.1% increase in the frequency of participants with hyperopia, while the frequency of those with myopia and emmetropia decreased by 5.5% and 23.3%, respectively. The average spherical equivalent difference (mean ± SD) induced by cycloplegia was 0.72 ± 0.73 D, with the highest difference observed in the 3–5 years age group (1.18 ± 0.85 D). The differences in astigmatism power (*p* = 0.84) and astigmatism axis (*p* = 0.97) between non-cycloplegic and cycloplegic conditions were not statistically significant.

## 1. Introduction

Eye refraction can be influenced by the accommodative status of the patient, especially in children and patients with high accommodative tonus. To accurately determine the refractive error of the eye, it is therefore necessary to relax the patient’s accommodation, either by optical methods, such as the fogging technique, or by pharmacological methods, such as cycloplegia [1]. In Latvia, optical fogging is the standard procedure used by optometrists, who typically employ a plus lens monocularly to reduce visual acuity to 0.2 (in decimal units) and then gradually reduce the fogging lens power [1], whereas cycloplegia is typically performed by ophthalmologists. It has been demonstrated that the fogging technique can reveal significantly lower plus values than refraction with cycloplegia [2], leading to biased classifications of refractive errors [3,4]. Several authors emphasize that cycloplegic refraction is essential for preventing the misclassification of refractive errors in children [3,4,5]; however, less focus has been placed on how children experience this procedure. For example, among children of Asian origin aged 2 to 12 years, 26% experienced significant pre-drop distress, and 13% were uncooperative with drops, with uncooperative children being more likely to be younger, male, and have a previous negative eyedrop experience [6]. The most common adverse side effects of cycloplegic eye drops include stinging upon instillation, blurred near vision, and light sensitivity [3,6,7]. Combined with longer appointment times, these factors call for careful consideration of the indication for cycloplegia to reduce the waste of medical resources, promote diagnostic efficiency, and improve the patient experience, without losing relevant clinical information provided by cycloplegia [6,8]. Globally, cycloplegic refraction is accepted as a standard procedure for assessing refractive errors in pediatric eye examinations and is the gold standard for epidemiological studies [9,10]. Clinical studies have highlighted the importance of cycloplegia for correct refractive error estimation, revealing latent hyperopia and avoiding the overestimation of myopia [11,12]. Careful selection of a cycloplegic procedure regimen (including agent, dose, and interval) should be tailored according to ethnic origin because the absorption of cycloplegic agents decreases in individuals with darker irises [13,14]; therefore, achieving sufficient cycloplegia may be more challenging, necessitating higher concentrations of cycloplegic agents or more drops [15]. The majority of previous studies evaluating changes in eye refraction after cycloplegia have been conducted in Asia [3,4,5,16,17]. It has been noted that individuals with lighter irises achieve deeper cycloplegia with lower dosages [18]; therefore, caution should be exercised to avoid unnecessarily high dosages of cycloplegics in individuals of European descent with light irises.

This study aims to investigate the objective refraction status before and after cycloplegia from childhood to young adulthood within the Latvian population to evaluate how age and non-cycloplegic types of refractive error influence the frequency of eye refractive errors, as well as the relationship between age and cycloplegia-induced changes in the power of refractive error.

## 2. Materials and Methods

### 2.1. Participants

**Figure 1:**
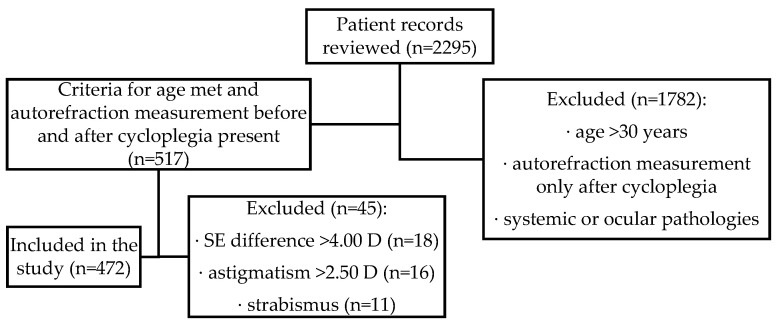
Flowchart of the patient record review process representing participant selection.

In total, 2295 patient records from a private ophthalmologist’s practice in Latvia were reviewed. From this dataset, 517 eye examinations performed with cycloplegia were selected, focusing on patients aged below 30 years. Patients with strabismus (assessed with the unilateral cover test), astigmatism > 2.50 D before or after cycloplegia, or suspected instrument myopia, reflected as a spherical equivalent (SE) change after cycloplegia > 4.00 D, were excluded from the sample. A total of 472 non-population-based patient records were selected for this study (see Figure 1), representing healthy participants of European origin aged between 3 and 28 years (mean age 9.1 ± 4.6 years) who attended a private ophthalmology practice either for state-funded regular eye examinations (at ages 3 years and 6 to 7 years) or for other unspecified reasons. 

The 472 participants, consisting of 260 females (55%) and 212 males (45%), were divided into six age groups, with demographic characteristics detailed in Table 1. There was no significant difference in mean age between genders (Z = −1.39, *p* = 0.16).

Cycloplegia was induced by instilling one drop of cyclopentolate 1% into the conjunctival sac of each eye, and autorefractor measurements were performed 30 ± 5 min after the instillation of 1% cyclopentolate. For this study, we used patient age and autorefraction measurements (both before and after cycloplegia) obtained from records at a private ophthalmology practice, covering examinations conducted between January 2018 and December 2020. The study was conducted in accordance with the Declaration of Helsinki and approved by the Scientific Research Ethics Committee of the Institute of Cardiology and Regenerative Medicine at the University of Latvia; approval No. 41/2020 (Date 25 February 2020). No identifiable data was included. 

### 2.2. Data Analysis

The spherical equivalent (SE) was calculated using the spherical power (Sph) and cylindrical power (Cyl) obtained from objective autorefraction measurements (SE = Sph + Cyl/2). To estimate the frequency of refractive errors before and after cycloplegia, we followed the commonly used cut-off points in epidemiological research [19]. Participants were categorized into groups based on SE values obtained from the autorefractometer: emmetropia (SE from >−0.50 to +0.50 D), hyperopia (SE > +0.50 D), myopia (SE ≤ −0.50 D), and astigmatism (any SE indicating simple hyperopic, simple myopic astigmatism, or mixed astigmatism). Participants with compound hyperopic or myopic astigmatism, based on SE calculations, were included in the hyperopia and myopia groups, respectively. For objective refraction values with a cylindrical power of 0.25 D only spherical power was analyzed; values with a cylindrical power of 0.50 D were directly converted to SE; and values with a cylindrical power of >0.50 D were classified as astigmatism and allocated to either the myopia or hyperopia group (in cases of compound astigmatism) or to the astigmatism group. In the astigmatism group, the astigmatism axis was categorized as with-the-rule (negative cylinder axis 180° ± 30°), against-the-rule (negative cylinder axis 90° ± 30°), or oblique (negative cylinder axis between 31° and 59° or 121° and 149°). 

Since the SE values in the right and left eyes were highly correlated (non-cycloplegic r_s_ = 0.86, *p* < 0.01 and cycloplegic r_s_ = 0.94, *p* < 0.01), we analyzed the data for the left eye only (in the non-cycloplegic condition, SE in the right eye was 0.14 D more myopic than in the left eye (the right eye was always measured first), and this difference decreased by half after cycloplegia, suggesting that for the left eye, the autorefractometer fogging managed to control accommodation better). 

### 2.3. Statistical Analysis

Statistical analysis was performed using SPSS (version 22.0; IBM Corporation, Armonk, NY, USA), visualizations were made in Microsoft Excel (365). The spherical equivalent (SE) of autorefraction values under non-cycloplegic and cycloplegic conditions exhibited non-normal distributions (Kolmogorov–Smirnov test, *p* < 0.05). Therefore, non-parametric tests were employed for analysis: the Wilcoxon Signed Ranks Test for comparing dependent data (specifically between non-cycloplegic and cycloplegic conditions), the Kruskal–Wallis H-test, and the Mann–Whitney U-test were used for comparing independent data (stratified by age, non-cycloplegic refractive status, and gender). Spearman’s rank coefficient was used to assess correlations. A significance level of *p* < 0.05 was applied.

## 3. Results

### 3.1. Frequency of Refractive Errors before and after Cycloplegia

**Figure 2:**
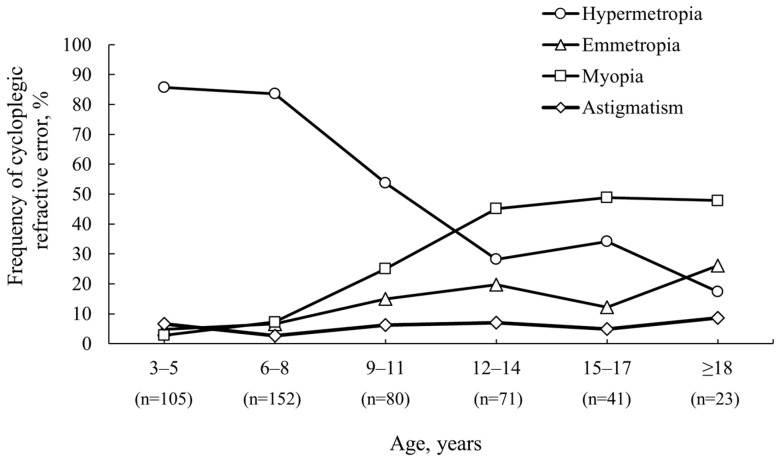
Frequency of cycloplegic eye refractive status stratified by age.

After cycloplegia, the overall frequency of participants with hyperopia increased by 34.1%, and the frequency of participants with myopia and emmetropia decreased by 5.5% and 23.3%, respectively. SE after cycloplegia revealed that the predominant refractive error among the study population of children attending ophthalmology practice (see Figure 2) aged 3–5 and 6–8 years was hyperopia (85.7% and 83.6%, respectively), which gradually decreased with age. The frequency of hyperopia was 53.8%, 28.2%, and 34.1% at ages 9–11, 12–14, and 15–17 years, respectively. From ages 12 to 14 years (45.1%), the predominant refractive error became myopia, maintaining its frequency in older age groups. The frequency of myopia was 48.8% and 47.8% at ages 15–17 and ≥18 years, respectively.

**Figure 3:**
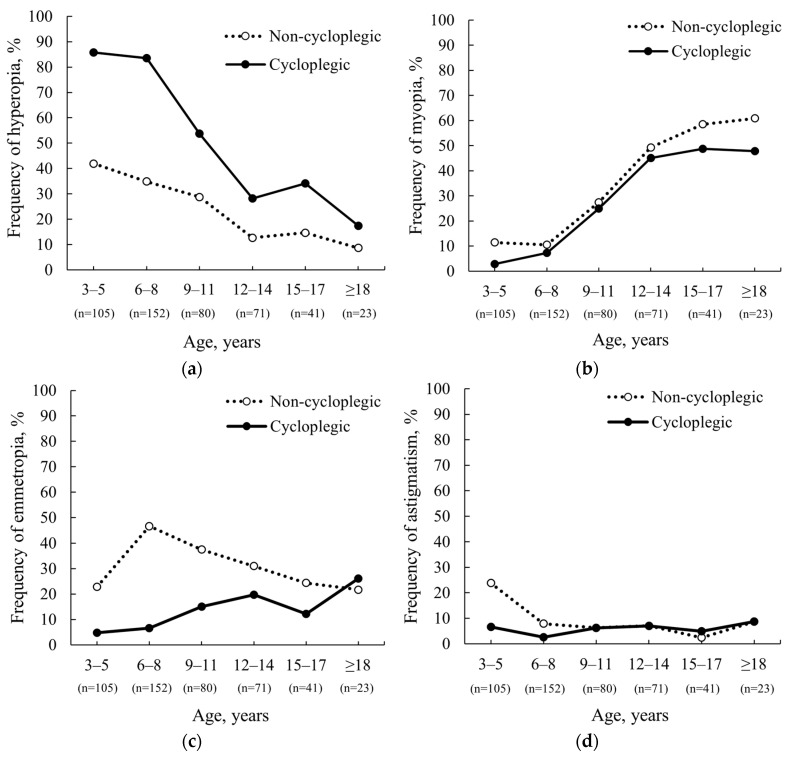
Frequency of refractive errors in different age groups before (dotted line: non-cycloplegic) and after (solid line: cycloplegic) cycloplegia: (**a**) Frequency of hyperopia, (**b**) Frequency of myopia, (**c**) Frequency of emmetropia, and (**d**) Frequency of astigmatism.

A relationship was found between age and the percentage of participants for whom the refractive error type changed after cycloplegia (r_s_ = 0.73, *p* = 0.002), highlighting the clinical value of cycloplegia, especially in younger children. A change in refractive error type after cycloplegia was observed in half of the participants aged 3–5 years (51.4%) and 5–8 years (49.3%), about one-third of participants aged 9–17 years (33.8% for 9–11 years, 29.6% for 12–14 years, and 26.8% for 15–17 years), and one-fifth of participants aged 18 years or older (21.7%). Our results demonstrate that non-cycloplegic autorefraction results underestimate the prevalence of hyperopia, especially in younger participants (see Figure 3a); underestimate the prevalence of emmetropia, except in participants aged 18 and above, where it is overestimated before cycloplegia (see Figure 3c); overestimate the prevalence of myopia, especially in older participants (see Figure 3b); and overestimate the prevalence of astigmatism only in younger participants (see Figure 3d).

Table 2 details changes in refractive error type distribution (Δ) across age groups induced by cycloplegia, along with the frequency of clinically significant SE changes (≥0.50 D).

### 3.2. Spherical Equivalent (SE) Difference after Cycloplegia

**Figure 4:**
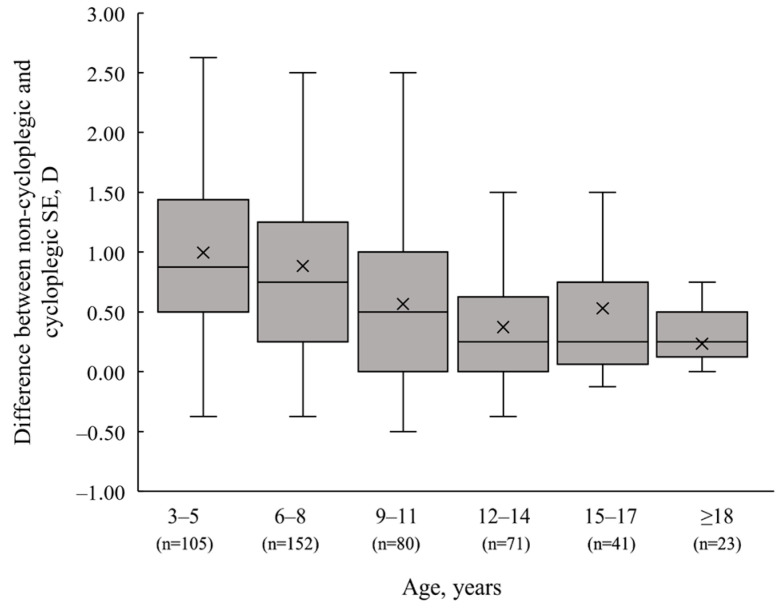
Difference between non-cycloplegic and cycloplegic condition determined by spherical equivalent (SE) change stratified by age group. The mean SE difference after cycloplegia is shown as ×, and the median is represented as a line inside the box. The whiskers extend from the top of the box (Q3) to the largest data point ≤ 1.5 times the IQR and from the bottom of the box (Q1) to the smallest data point > 1.5 times the IQR.

The mean cycloplegic SE of 0.71 ± 2.03 D (95% CI: 0.52 to 0.89 D) showed a statistically significant hyperopic shift (*p* < 0.01) compared to the non-cycloplegic SE of −0.01 ± 1.74 D (95% CI: −0.17 to 0.15 D). The average SE difference (mean ± SD) induced by cycloplegia was 0.72 ± 0.73 D (95% CI: 0.65 to 0.78 D). Overall, cycloplegia induced a clinically significant (≥0.50 D) hyperopic shift in SE in 60.2% of participants, a myopic shift in 1%, a clinically minor shift (<0.50 D) in 27.1% and no shift in 11.7%. The difference in SE between non-cycloplegic and cycloplegic refraction was significantly associated with younger age (r_s_ = −0.36; *p* < 0.01), indicating that cycloplegia is a valuable diagnostic technique for younger children to reveal latent refractive errors that can be masked by active accommodation in non-cycloplegic autorefraction. For children aged 3 to 5 years and 6 to 8 years, the SE change after cycloplegia was higher (mean (median) ± SD (IQR): 1.00 (0.88) ± 0.73 (0.94) D and 0.88 (0.75) ± 0.74 (1.00) D, respectively) compared to children aged 12 years and older (see Figure 4 and Table 3).

When SE difference was stratified by non-cycloplegic refractive error status, the mean SE difference after cycloplegia was significantly lower for participants with myopia (mean (median) ± SD (IQR): 0.45 (0.25) ± 0.79 (0.50) D, 95% CI: 0.31 to 0.59 D) compared to participants with hyperopia (0.73 (0.63) ± 0.71 (0.75) D 95% CI: 0.61 to 0.85 D, *p* < 0.01), emmetropia (0.85 (0.75) ± 0.66 (1.00) D, 95% CI: 0.75 to 0.95 D, *p* < 0.01), or astigmatism (0.89 (0.81) ± 0.72 (1.00) D, 95% CI: 0.69 to 1.00 D, *p* < 0.01). The SE difference after cycloplegia was not related to gender (mean (median) ± SD (IQR): 0.69 (0.50) ± 0.73 (0.84) D for females and 0.75 (0.56) ± 0.74 (1.00) D for males (Z = −0.65, *p* = 0.52)).

### 3.3. Changes in Astigmatism Power and Axis after Cycloplegia

**Figure 5:**
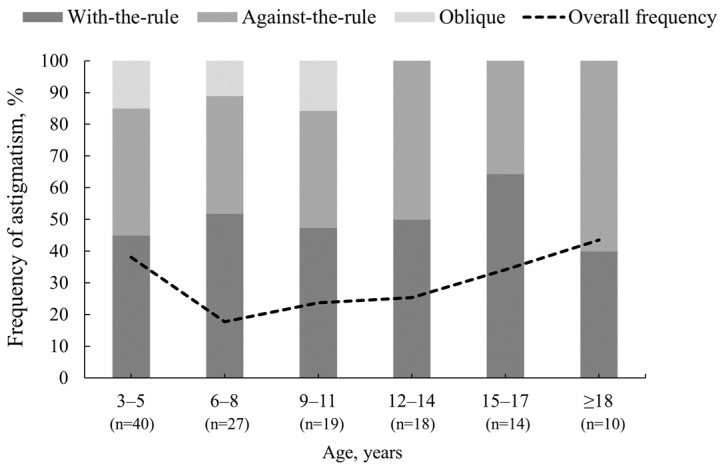
The total occurrence of clinically significant astigmatism (>0.50 D) is represented by a dashed line (n = 128, 27%). The 100% stacked column chart displays the frequency of the clinically significant astigmatism axis in the cycloplegic condition stratified by age.

Of the 472 participants, 36% (n = 172) exhibited clinically significant astigmatism > 0.50 D before and/or after cycloplegia. The total occurrence of astigmatism was higher for participants aged 3–5 years and ≥18 years (40% and 43%, respectively), while the lowest rate of astigmatism was observed for participants aged 6–8 years (18%) (see Figure 5). Clinically significant astigmatism observed under both non-cycloplegic and cycloplegic conditions was found in 19% of participants; for 10%, the astigmatism remained the same before and after cycloplegia, while, for 9%, cycloplegia induced insignificant changes in the negative astigmatism axis: 13° ± 17° counterclockwise in 24 participants and 12° ± 18° clockwise in 20 participants (*p* = 0.97). Before cycloplegia, astigmatism of >0.50 D was present in 134 participants (28%). After cycloplegia, astigmatism disappeared or decreased to ≤0.50 D in 9% of participants (33 participants had non-cycloplegic astigmatism of 0.75 D, for 8 participants it was 1.00 D, and for 3 participants it was 1.25 D), while new astigmatism > 0.50 D appeared in 8% of participants and did not exceed 1.25 D. Overall, within the study population the difference in astigmatism power between non-cycloplegic and cycloplegic conditions was not statistically significant (*p* = 0.84).

## 4. Discussion

In the current study, the frequency of hyperopia after cycloplegia increased by approximately one-third (34.1%), the frequency of myopia decreased slightly (5.5%), and the frequency of emmetropia decreased by approximately one-fifth (23.3%). Our results partially align with a study conducted by Zhao et al. (2004) in China, where participants aged 7–18 years who underwent cycloplegia, induced by two drops of 1% cyclopentolate administered 5 min apart, showed only a 12.7% increase in hyperopia and a considerably higher 55.9% decrease in myopia after cycloplegia [3]. A more recent study conducted on the Tibetan Plateau, China by Li et al. (2021) that involved first-grade students (mean age: 6.83 ± 0.46 years) found that after cycloplegia, the prevalence of myopia and emmetropia decreased by 10.66% and 35.9%, respectively, while the prevalence of hyperopia increased by 46.56% [20]. This aligns well with our results when compared with the 6 to 8 years age group in our study, where we observed 3.3% decrease in myopia, a 40.1% decrease in emmetropia, and a 48.7% increase in hyperopia. The relevance of cycloplegia is crucial when evaluating the epidemiological prevalence of refractive errors in children, as the present study demonstrated a strong relationship between age and the likelihood of the type of refractive error changing after performing cycloplegia (r_s_ = 0.73, *p* = 0.002). Non-cycloplegic autorefraction results tend to underestimate the prevalence of hyperopia, especially in younger children, and overestimate the prevalence of myopia, particularly in young adults, making these results unsuitable for epidemiological studies in these populations. While autorefraction data is not directly used to prescribe glasses and, therefore, the absence of cycloplegia does not necessarily impact prescription accuracy, non-cycloplegic autorefraction still serves as an approximation of expected refraction and can influence clinical decisions, such as the strategy of subjective correction when choosing the power for the fogging technique.

The refractive changes observed in our study are consistent with previous studies concluding that the cycloplegia procedure is a valuable diagnostic tool for accurately estimating full eye refractive status, thereby avoiding the overestimation of myopia and the underestimation of hyperopia [3,4,5,16,20]. Our study demonstrated that for younger children of European origin aged 3–8 years, changes in SE after cycloplegia were significantly larger (*p* < 0.05) than those in participants aged 9–18 years. A similar trend, emphasizing the need for cycloplegia procedures in preschool children, has been demonstrated in studies performed in Asia [4,8,12,16,20].

The current study also showed that the non-cycloplegic refractive error status influences the extent of the changes in SE after cycloplegia. For participants with non-cycloplegic myopia, the mean SE change was significantly lower (0.45 ± 0.79 D) compared to SE changes for participants with hyperopia (0.73 ± 0.71 D), emmetropia (0.85 ± 0.66 D), or astigmatism (0.89 ± 0.72 D). Jin et al. (2021) observed a mean SE change of 0.60 ± 0.55 D in Chinese children aged 4 to 15 years, with observations aligning with our results—significantly larger SE changes in participants with hyperopia (1.12 ± 0.64 D) compared to those with emmetropia (0.56 ± 0.43 D, *p* < 0.01) or myopia (0.32 ± 0.28 D, *p* < 0.01) [8]. Zhao et al.’s (2004) study, involving participants aged 7 to 18 years, found a larger SE change after cycloplegia in participants with moderate to high hyperopia (SE difference, mean ± SD: 2.98 ± 1.65 D) [3]. For participants aged 7 to 18 years with myopia of at least −2.00 D, they reported an SE difference of 0.41 ± 0.46 D, which is very similar to the difference observed in the current study (SE difference: 0.45 ± 0.79 D), despite the fact that we used one drop of cyclopentolate 1% for all participants, while Zhao et al. (2004) used 2 drops of cyclopentolate 1% for participants aged 7 to 18 years [3]. Li et al. (2021) found a mean difference in SE between cycloplegic and non-cycloplegic refraction of 0.90 ± 0.76 D in first-grade students [20], which closely aligns with the observed difference of 0.88 ± 0.74 D in our study group of 6- to 8-year-olds. Sanfilippo et al. (2014) and Bagheri et al. (2018) studied older participants aged 13 to 26 years and 10 to 40 years, respectively [17,21]. Sanfilippo et al. (2014) reported a lower overall SE difference for those with myopia (0.11 ± 0.47 D), with a 0.23 ± 0.48 D change for participants aged 13 to 19 years and a 0.02 ± 0.45 D change for those aged 20 to 26 years [17]. Bagheri et al. (2018) reported an overall hyperopic shift of 0.40 ± 0.50 D [21] and, aligning with our results, they reported more significant hyperopic shift in participants with hyperopia compared to participants with myopia (0.90 ± 0.50 D and 0.40 ± 0.50 D). In our study, for participants aged 12 or older with myopia, the observed SE difference was 0.20 ± 0.47 D, which aligns well with the data from Sanfilippo et al. (2014) for participants aged 13 to 19 years. We had only six participants over 19 years old, with an average SE difference of 0.08 ± 0.39 D. Our results demonstrate that the SE difference after cycloplegia was not related to gender, consistent with findings from other similar studies [3,8,16].

Some studies have emphasized the importance of defining what can be considered a clinically significant change in refractive error, which has been defined as ≥0.50 D [8] or >0.50 D [21], to avoid unnecessary side effects and long waiting times when performing cycloplegia [8]. In study [21], which included 106 participants aged 10 to 40 years (mean ± SD: 28 ± 5 years), 28.8% experienced an SE change of more than 0.50 D after cycloplegia. We defined a clinically significant change as ≥0.50 D [8], and observed this in 73.5% of participants with emmetropia, 72.0% with astigmatism, 68.6% with hyperopia, and 32.5% with myopia. By age group, clinically significant SE changes were observed in 80% of participants aged 3 to 5 years, 69.9% of those aged 6 to 8 years, 55% of those aged 9 to 11 years, and 40.7% of those aged 12 years or older. Detailed results of the frequency of participants with clinically significant SE changes, stratified by age and refractive error type, are presented in Table 2.

Within the current study, the difference in astigmatism between non-cycloplegic and cycloplegic conditions was not statistically significant (*p* = 0.84). A study conducted in Iran [22] on participants aged 3–59 years who underwent cycloplegia with 1% cyclopentolate found significant changes in astigmatism only in hyperopic eyes, with no significant changes in those with emmetropia or myopia. The statistically significant change observed was limited to a small J_0_ vector shift (mean (IQR) ± SD: 0.01 (0) ± 0.12, *p* = 0.006), suggesting that this shift may not be clinically significant [22]. Zhao et al. (2004) observed clinically small but statistically significant differences between non-cycloplegic and cycloplegic measurements of astigmatism, with mean J_0_ differences of −0.08 ± 0.13 D and mean J_45_ differences of −0.01 ± 0.09 D [3]. These findings are consistent with our results, further supporting the conclusion that the observed changes in astigmatism under cycloplegic conditions are not clinically significant. It should be acknowledged that for participants with astigmatism greater than 2.50 D, further studies could be beneficial, as Li et al. (2021) found that grade one primary school students with astigmatism above 2.50 D exhibited a greater difference in SE after cycloplegia compared to those with lower levels of astigmatism (*p* < 0.05) [20].

Additionally, it is important to consider differences in cycloplegic agent regimens when interpreting the observed changes between studies mentioned above. Sanfilippo et al. (2014) used one drop of cyclopentolate 1% for participants aged 13 to 14 years and one drop of tropicamide 1% for participants aged 15 to 26 years [17], Asharlous et al. (2016) used cyclopentolate 1% for participants aged 3–59 years [22], Zhao et al. (2004) and Bagheri et al. (2018) used 2 drops of cyclopentolate 1% for participants aged 7 to 18 years and 10 to 40 years, respectively [3,21], Li et al. (2021) achieved cycloplegia with 2 drops of cyclopentolate 1% and 1 drop of Mydrin P at a 5-min interval [20]. In our study, we used one drop of cyclopentolate 1% for participants aged 3 to 28 years. It should be noted that the cycloplegia procedure using a single drop of cyclopentolate 1% has been empirically shown to be effective and sufficient for most individuals of European origin [15]. In contrast, studies involving Asian or African American individuals with darker iris colors often require multiple drops of cycloplegic agents. For instance, Zhu et al. (2016) [5] found that for 41% of participants aged 6 to 21 years from Inner Mongolia, China, 2 drops of cyclopentolate 1% administered five minutes apart were insufficient to dilate pupils beyond 6 mm. Eye care providers should be aware that, in specific cases, patients of European origin may require a second drop of cyclopentolate 1% to achieve full cycloplegia. Sanfilippo et al. (2014) concluded that over the age of 20, the difference between non-cycloplegic and cycloplegic refraction is insignificant, suggesting that the cycloplegia procedure should be performed for children and young adults below 20 years of age [17].

Various studies have demonstrated the importance of cycloplegia during comprehensive eye examinations in young individuals. The selection of participants in these studies, including their ethnic origin, age, and whether they were from a population-based or non-population-based sample, as well as the cycloplegic regimen used (including the type and dosage of the agent and the interval between doses), significantly affects the amount of change in spherical equivalent (SE) and the frequency of refractive error after cycloplegia.

### 4.1. Study Limitations

Participants recruited for this study attended ophthalmology practice for reasons unrelated to the current study. Therefore, it is crucial to acknowledge that our study population was biased, as it included participants predisposed to ocular-related dysfunction, except for those aged 3 years and 6–7 years, who attended regular eye examinations. Consequently, the refractive error frequencies found in our study population did not directly reflect the situation in the Latvian population. Currently, there are no official data available regarding the prevalence of myopia in Latvian children. We found that from 12 to 14 years of age, myopia was the predominant refractive error after cycloplegia. However, this should be interpreted cautiously, considering that the study population was not representative of the general population and likely included individuals attending eye examinations due to subjective complaints. A comprehensive review encompassing 28 cross-sectional studies published between January 2013 and March 2019 reported the prevalence of myopia in schoolchildren globally [23]. Only three of these studies were conducted in Europe. In Denmark, the prevalence of myopia in children aged 5–10 years was estimated to be 17.9% [24]. In France, the prevalence of myopia was 19.6% among children aged 0–9 years and 42.7% among those aged 10–19 years [25]. In the Netherlands, a study of 5711 six-year-old children estimated the prevalence of myopia to be 2.4%. The average prevalence of myopia in children in Asia was significantly higher than in European children (60% compared to 40%) [23]. 

### 4.2. Local Context

Optometrists in Latvia obtain a five-year education, comprising a three-year bachelor’s degree and a two-year master’s degree, to become certified clinical optometrists. From January 2020, Latvian optometrists have been registered as medical practitioners who, therefore, work in accordance with the local Medical Treatment Law issued by the Republic of Latvia. Currently, state-funded eye examinations for children are available in Latvia by ophthalmologists. Regulation No. 555 of the Cabinet of Ministers of the Republic of Latvia mandates that three state-funded preventive eye examinations for children must be performed by an ophthalmologist at the ages of 13–24 months, 3, and 6–7 years. Each of these examinations must include an objective refraction measurement with cycloplegia. Optometrists in Latvia participate in vision screening programs or offer paid pediatric eye examinations in optic stores without cycloplegia. To avoid overcorrecting myopia, Latvian optometrists perform subjective correction using the fogging technique, adhere to the rule of maximum plus and minimum minus lenses that provide the best correction for visual acuity, use the duochrome test (where better clarity on green indicates potential overcorrection of myopia or undercorrection of hyperopia), ensure that negative relative accommodation does not exceed +2.50 D at 40 cm, and consider near esophoria as a potential indicator of overcorrection of myopia or latent hyperopia. Given the alarming decline in the number of residents pursuing pediatric ophthalmology in the U.S. [26] and similar trends observed in Latvia, involving optometrists more extensively in pediatric care could be a crucial solution to improving access to eye care, as they are well-equipped to manage many aspects of pediatric optometry and can help address the growing shortage of pediatric ophthalmologists.

## 5. Conclusions

The study results demonstrate that cycloplegia significantly alters refractive error, revealing a clinically significant (≥0.50 D) hyperopic shift in SE for 60.2% of participants, a myopic shift in 1%, a clinically minor shift (<0.50 D) in 27.1%, and no shift in 11.7%, resulting in a 34.1% increase in the frequency of participants with hyperopia, while the frequency of those with myopia and emmetropia decreased by 5.5% and 23.3%, respectively. The average spherical equivalent (SE) difference (mean ± SD) induced by cycloplegia was 0.72 ± 0.73 D. Changes in SE were most pronounced in younger age groups, highlighting the value of cycloplegia in uncovering latent hyperopia during pediatric vision examinations and in estimating refractive error types in epidemiological studies. The differences in astigmatism power and axis between non-cycloplegic and cycloplegic conditions were not statistically significant, suggesting that cycloplegia primarily affects spherical refractive components.

## Figures and Tables

**Table 1:** Demographic characteristics of the study population.

	Participants, n (%)	Age, Years (Mean (Median) ± SD (IQR))
Age groups (years):		
3–5 years (n = 105)	105 (22%)	3.8 (3.7) ± 0.7 (1.0)
6–8 years (n = 152)	152 (32%)	7.0 (7.0) ± 0.6 (0.7)
9–11 years (n = 80)	80 (17%)	9.9 (9.9) ± 0.9 (1.6)
12–14 years (n = 71)	71 (15%)	13.0 (12.8) ± 0.8 (1.4)
15–17 years (n = 41)	41 (9%)	15.9 (16.0) ± 0.9 (1.7)
≥18 years (n = 23)	23 (5%)	20.2 (19.2) ± 3.0 (3.9)
Gender:		
Female	260 (55%)	9.4 (7.9) ± 4.7 (6.4)
Male	212 (45%)	8.8 (7.3) ± 4.5 (5.3)
Total:	472 (100%)	9.1 (7.7) ± 4.6 (6.1)

**Table 2:** Frequency of refractive error type distribution across age groups under non-cycloplegic and cycloplegic conditions, including changes in frequency distribution (Δ) and frequency of clinically significant spherical equivalent (SE) changes (≥0.50 D) after cycloplegia.

	Frequency, n (%), Non-Cycloplegic	Frequency, n (%), Cycloplegic	Change in Frequency ∆, n (%)	Clinically Significant Changes in SE, n (%)
**3–5 years**	105 (100)			84 (80.0)
Hyperopia (n = 44)	44 (41.9)	90 (85.7)	46 (43.8)	32 (72.8)
Myopia (n = 12)	12 (11.4)	3 (2.9)	9 (8.5)	12 (100)
Emmetropia (n = 24)	24 (22.9)	5 (4.8)	19 (18.1)	20 (83.3)
Astigmatism (n = 25)	25 (23.8)	7 (6.7)	18 (17.1)	20 (80.0)
**6–8 years**	152 (100)			106 (69.7)
Hyperopia (n = 53)	53 (34.9)	127 (83.6)	75 (48.7)	32 (60.4)
Myopia (n = 16)	16 (10.5)	11 (7.2)	5 (3.3)	6 (37.5)
Emmetropia (n = 71)	71 (46.7)	10 (6.6)	61 (40.1)	60 (84.5)
Astigmatism (n = 12)	12 (7.9)	4 (2.6)	8 (5.3)	8 (66.7)
**9–11 years**	80 (100)			44 (55.0)
Hyperopia (n = 23)	23 (28.7)	43 (53.8)	20 (25.1)	18 (78.3)
Myopia (n = 22)	22 (27.5)	20 (25.0)	2 (2.5)	4 (18.2)
Emmetropia (n = 30)	30 (37.5)	12 (15.0)	18 (22.5)	19 (63.3)
Astigmatism (n = 5)	5 (6.3)	5 (6.3)	0 (0)	3 (60.0)
**≥12 years**	135 (100)			55 (40.7)
Hyperopia (n = 17)	17 (12.6)	38 (28.1)	21 (15.5)	12 (70.6)
Myopia (n = 73)	73 (54.1)	63 (46.7)	10 (7.4)	18 (24.7)
Emmetropia (n = 37)	37 (27.4)	25 (18.5)	12 (8.9)	20 (54.1)
Astigmatism (n = 8)	8 (5.9)	9 (6.7)	1 (0.8)	5 (62.5)
**Total**	472 (100)			289 (61.2)
Hyperopia (n = 137)	137 (29.0)	298 (63.1)	161 (34.1)	94 (68.6)
Myopia (n = 123)	123 (26.1)	97 (20.6)	26 (5.5)	40 (32.5)
Emmetropia (n = 162)	162 (34.3)	52 (11.0)	110 (23.3)	119 (73.5)
Astigmatism (n = 50)	50 (10.6)	25 (5.3)	25 (5.0)	36 (72.0)

Δ—difference between non-cycloplegic and cycloplegic SE, D—diopter.

**Table 3:** Mean ± standard deviation (SD), median (IQR) and 95% confidence interval (CI) of spherical equivalent (SE) in different age groups under non-cycloplegic, cycloplegic conditions, and difference between non-cycloplegic and cycloplegic condition (Δ).

	Mean SE ± SD, D	Median (IQR), D	95% CI
**3** **–** **5 years (n = 105)**			
Non-cycloplegic	0.37 ± 0.82	0.50 (1.06)	0.21 to 0.53
Cycloplegic	1.37 ± 0.81	1.50 (0.75)	1.21 to 1.52
Δ	1.00 ± 0.73	0.88 (0.94)	0.86 to 1.14
**6** **–** **8 years (n = 152)**			
Non-cycloplegic	0.42 ± 1.23	0.44 (0.75)	0.22 to 0.61
Cycloplegic	1.30 ± 1.43	1.25 (1.00)	1.07 to 1.53
Δ	0.88 ± 0.74	0.75 (1.00)	0.77 to 1.00
**9** **–** **11 years (n = 80)**			
Non-cycloplegic	0.14 ± 2.01	0.25 (1.47)	−0.30 to 0.59
Cycloplegic	0.71 ± 2.27	0.75 (2.09)	0.21 to 1.22
Δ	0.57 ± 0.63	0.50 (1.00)	0.43 to 0.71
**12** **–** **14 years (n = 71)**			
Non-cycloplegic	−0.77 ± 1.80	−0.50 (1.63)	−1.20 to −0.35
Cycloplegic	−0.40 ± 1.97	−0.12 (2.13)	−0.87 to 0.07
Δ	0.37 ± 0.57	0.25 (0.63)	0.24 to 0.51
**15** **–** **17 years (n = 41)**			
Non-cycloplegic	−0.99 ± 2.59	−0.62 (3.00)	−1.80 to −0.17
Cycloplegic	−0.46 ± 3.23	−0.50 (3.63)	−1.47 to 0.56
Δ	0.53 ± 0.87	0.25 (0.69)	0.26 to 0.81
**≥18 years (n = 23)**			
Non-cycloplegic	−1.03 ± 2.73	−0.75 (3.38)	−2.21 to 0.15
Cycloplegic	−0.80 ± 2.89	−0.25 (3.25)	−2.05 to 0.45
Δ	0.23 ± 0.31	0.25 (0.38)	0.10 to 0.37
**Total (n = 472)**			
Non-cycloplegic	−0.01 ± 1.74	0.25 (1.25)	−0.17 to 0.15
Cycloplegic	0.71 ± 2.03	1.00 (1.59)	0.52 to 0.89
Δ	0.72 ± 0.73	0.50 (1.00)	0.65 to 0.78

SE—spherical equivalent, SD—standard deviation, D—diopter, IQR—interquartile range, Δ—difference between non-cycloplegic and cycloplegic, 95% CI—95% confidence interval.

## Data Availability

Data are available on request from the corresponding author.
